# Improving Quality of End-of-Life Care Through the K-HOPE Consultative Palliative Care Model: A Prospective Study in a Tertiary Hospital

**DOI:** 10.3390/curroncol33040213

**Published:** 2026-04-13

**Authors:** Yoo Jeong Lee, In Cheol Hwang, Eun Jeong Lee, Soon-Young Hwang, Youn Seon Choi

**Affiliations:** 1 Palliative Care Center, Korea University Guro Hospital, Seoul 08308, Republic of Korea; juujeong@naver.com (Y.J.L.); 282lej@kumc.or.kr (E.J.L.); 2 Department of Family Medicine, Korea University Guro Hospital, Seoul 08308, Republic of Korea; 3 Department of Family Medicine, Gil Medical Center, Gachon University College of Medicine, Incheon 21936, Republic of Korea; spfe0211@gmail.com; 4 Department of Biostatistics, Korea University College of Medicine, Seoul 02841, Republic of Korea; hwangsy214@gmail.com

**Keywords:** consultative palliative care, end-of-life care, palliative care, patient reported outcome measures, quality of life, terminal cancer

## Abstract

As population aging accelerates, the demand for high-quality end-of-life (EOL) care continues to rise. However, a substantial proportion of patients with terminal cancer still experience death in acute-care hospitals without adequate palliative care. Consultative palliative care (CPC) represents a feasible model for delivering palliative care without requiring dedicated inpatient units, yet evidence evaluating its clinical impact remains limited. In this study, we developed a structured hospital-based CPC model tailored to the Korean healthcare system, the Korea Holistic Optimized Palliative care for End-of-life (K-HOPE) model, and prospectively evaluated its clinical impact. K-HOPE was delivered by an interdisciplinary CPC team in a tertiary hospital. Unmet needs were assessed using the Integrated Palliative care Outcome Scale (IPOS), and longitudinal changes were analyzed using mixed-effects models for repeated measures. Among patients who died during hospitalization, quality of death was evaluated using the Good Death Scale (GDS). A total of 84 patients with terminal cancer received K-HOPE. The total IPOS score significantly decreased over time (β = −10.4, 95% CI −12.8 to −8.0; *p* < 0.001), indicating reduced overall burden and unmet needs. Significant improvements were observed in psychological distress (*p* = 0.010) and communication and information needs (*p* < 0.001), whereas changes in physical symptoms and practical concerns were not statistically significant. Among 22 patients who died during hospitalization, 59.1% achieved a good quality of death (GDS ≥ 12). Longer duration of CPC involvement was significantly associated with higher quality of death and remained an independent predictor in multivariable analysis. These findings suggest that the K-HOPE CPC model improves communication and overall EOL care experiences among hospitalized patients with terminal cancer, indicating that meaningful improvements in EOL care can occur even during short periods of CPC involvement. Structured CPC integrated into routine oncology practice represents a feasible strategy for improving EOL care in tertiary hospitals, and a standardized CPC framework may enhance the consistency and reproducibility of care delivery within the Korean healthcare system.

## Simple Summary

Patients with terminal cancer in Korea often receive end-of-life (EOL) care in acute-care hospitals, where limited time and fragmented care processes can make it difficult to address their complex needs. To address this challenge, we developed a structured consultative palliative care (CPC) model tailored to the Korean healthcare system, the Korea Holistic Optimized Palliative Care for End-of-Life (K-HOPE) model. In this study, we applied the K-HOPE model in a tertiary hospital and evaluated its effects on patients’ unmet needs and EOL care experiences. We found that this approach improved communication and reduced unmet needs among hospitalized patients with terminal cancer. Importantly, meaningful improvements in EOL care were observed even during relatively short periods of palliative care involvement. These findings highlight the potential role of structured CPC in supporting high-quality EOL care within routine oncology practice in tertiary hospitals, particularly by providing a standardized approach that may enhance consistency of care delivery within the Korean healthcare system.

## 1. Introduction

Population aging is accelerating worldwide, leading to a growing demand for comprehensive and high-quality end-of-life (EOL) care. In the Republic of Korea, the transition to a super-aged society is occurring at an exceptionally rapid pace, accompanied by an increasing prevalence of chronic and multi-morbid conditions among older persons [1]. In such contexts, disease-directed treatment alone is insufficient to address the complex constellation of physical, psychological, social, and existential needs experienced by patients at EOL [2]. This underscores the necessity of continuous, holistic, and person-centered EOL care that supports patients and families across multiple domains [3].

Despite this need, patients with terminal cancer in Korea frequently spend their final days in acute-care hospitals, where substantial unmet needs persist—particularly in symptom management, emotional support, communication, and decision-making [4]. According to the World Health Organization (WHO), high-quality EOL care requires an integrated interdisciplinary approach that encompasses physical, emotional, social, and spiritual elements of suffering [5]. However, in real-world acute-care settings where life-prolonging interventions are often prioritized, structural constraints, time pressure, and delayed palliative care referral commonly hinder the consistent delivery of such holistic care [6].

Consultative palliative care (CPC) has emerged internationally as a feasible model for delivering palliative care without requiring dedicated palliative care units [7]. Interdisciplinary palliative care teams provide expert consultation within general wards or outpatient settings, supporting symptom control, communication, goals-of-care discussions, and care transitions. Studies have shown that CPC improves symptom burdens, enhances patient and family satisfaction, reduces intensive or nonbeneficial treatments, and promotes more appropriate use of healthcare resources [8,9,10]. Nonetheless, in Korea, the structure and implementation of CPC vary widely across institutions, and empirical evidence evaluating its multidimensional impact remains limited. In particular, few studies have assessed changes in patients’ experiences in palliative care and end-of-life care using patient-reported outcome measures [11,12].

Palliative care is a broad, interdisciplinary approach that can be integrated throughout the disease trajectory, whereas EOL care refers specifically to care provided in the final stage of life. In this study, CPC is conceptualized as a model of palliative care delivery within acute-care settings, and its effects are evaluated in the context of EOL care among hospitalized patients. In addition, referral to palliative care in Korea is often delayed and occurs late in the disease trajectory, partly due to persistent misconceptions that palliative care is only for the imminently dying and limited awareness among both patients and healthcare professionals [13]. Furthermore, in Korea, CPC has only recently been implemented and is not yet fully integrated into routine clinical practice [14]. As a result, many patients receive palliative care at a very advanced stage of illness, with limited time for comprehensive intervention.

To address this gap, we developed the Korea Holistic Optimized Palliative care for End-of-life (K-HOPE) model, a standardized hospital-based CPC framework tailored to the Korean tertiary acute-care hospital setting. This study applied the K-HOPE CPC model in a real-world clinical setting and prospectively evaluated its effects on the unmet needs and care experiences of patients with terminal cancer. Specifically, we aimed to determine whether the K-HOPE CPC model, as a palliative care intervention, was associated with improvements in symptom burden, communication, and patient-reported outcomes related to EOL care among hospitalized patients with terminal cancer.

## 2. Materials and Methods

### 2.1. Study Design and Settings

This prospective observational study was conducted at Korea University Guro Hospital, a tertiary hospital in Korea. Participants were enrolled between January 2023 and January 2025. All study procedures were conducted within the routine clinical workflow of the hospital-based palliative care center.

Korea University Guro Hospital provides comprehensive acute and specialized care, including oncology, intensive care, and emergency services. During the study period, the palliative care center operated a CPC service, providing hospital-wide consultations through physician-initiated referrals. The CPC team, consisting of physicians, nurses, and social workers, primarily delivered consultative services to hospitalized patients with terminal cancer. Although non-cancer conditions such as chronic obstructive pulmonary disease (COPD) and liver cirrhosis are included as eligible diagnoses for palliative care in Korea, referrals for these conditions were extremely rare in our setting, typically accounting for only one to two cases per year.

**Figure 1:**
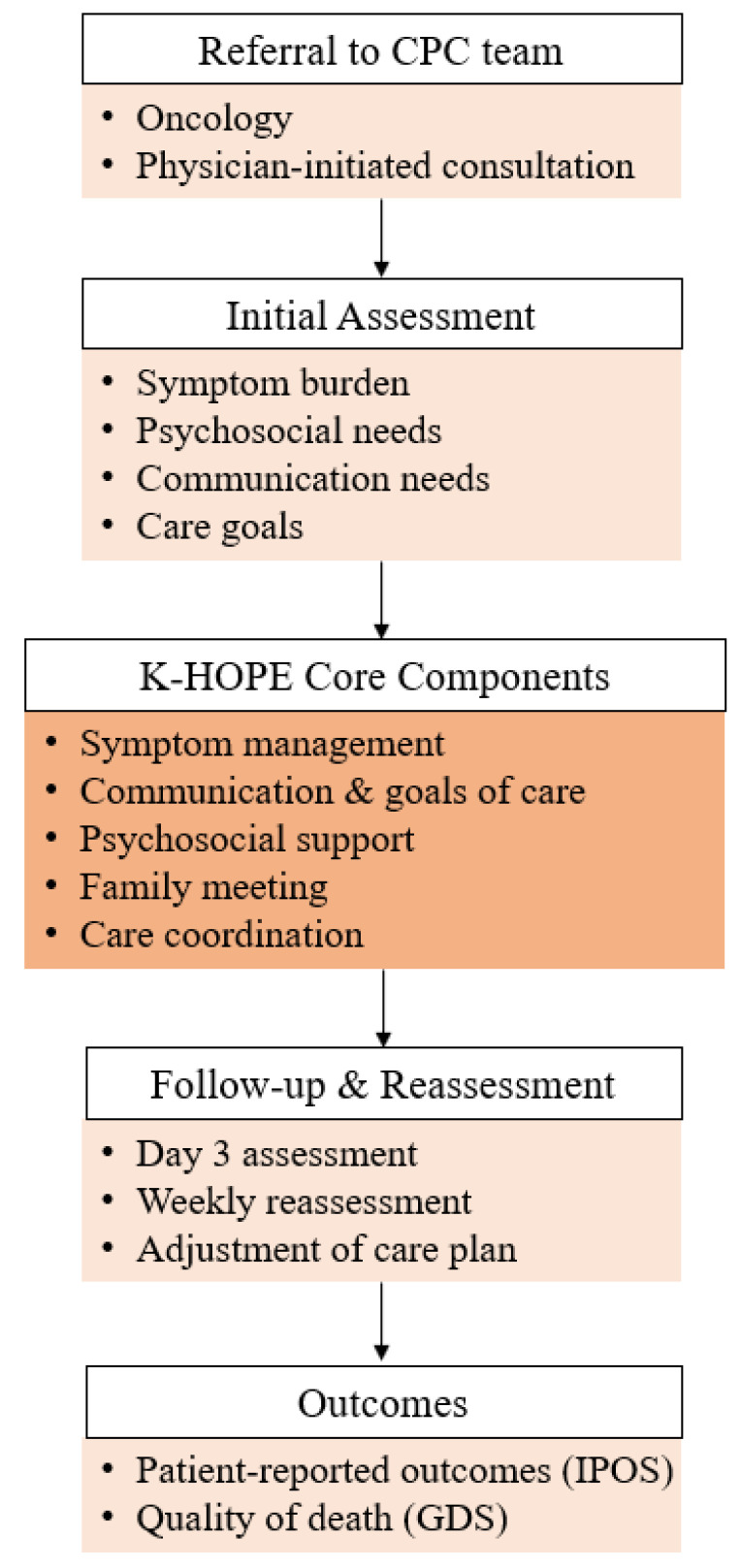
Standard workflow of the K-HOPE consultative palliative care model.

The K-HOPE model consists of a structured set of CPC components encompassing pre-counseling, holistic needs assessment, individualized care planning, multidisciplinary symptom management, psychosocial and spiritual support, and care transition planning across the EOL trajectory (Table 1). All clinical assessments and interventions were prospectively documented using standardized electronic medical record forms integrated into the hospital’s cloud-based Patient-Hospital Information System (P-HIS). A flow chart is presented to illustrate the standard application of the K-HOPE model, including referral, assessment, core interventions, and follow-up (Figure 1).

**Figure 2:**
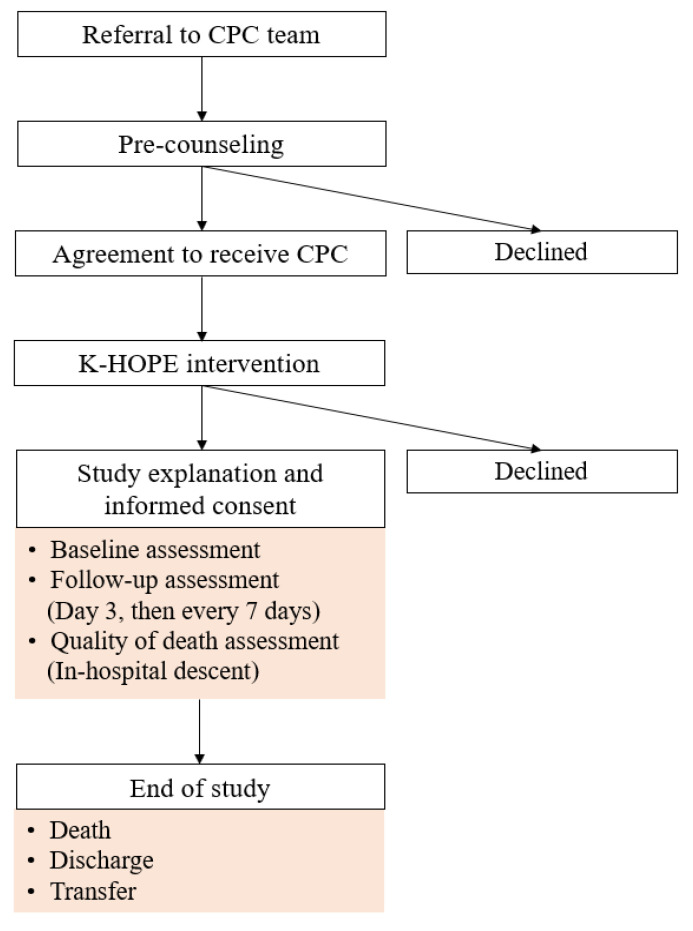
Study protocol and assessment schedule of the K-HOPE model.

Following referral to the CPC team, patients and families underwent a pre-counseling session. Patients who agreed to receive CPC subsequently received the K-HOPE as part of routine clinical care, regardless of study participation. Patients who provided informed consent for study participation were enrolled in the study. Informed consent was obtained from all participants prior to enrollment. Only patients who were able to understand the study and provide written informed consent were included, and those with impaired decision-making capacity were excluded. Baseline assessments of unmet needs were performed at the time of the initial CPC consultation. Follow-up assessments were conducted three days after the baseline and subsequently at seven-day intervals, based on a predefined study protocol rather than patient-initiated requests. In cases of clinical deterioration or changes in patient condition, additional assessments were performed at the discretion of the CPC team. For patients identified as being in the last days of life, based on institutional clinical criteria (including Karnofsky Performance Scale ≤ 20 and the presence of at least two signs such as markedly reduced oral intake, altered consciousness or increased sleep, terminal delirium, or dyspnea or death rattle), comfort care was implemented, and the quality of death was evaluated after death by the palliative care team. Patients were followed until CPC involvement ended due to death, discharge, or transfer, at which point study participation was concluded (Figure 2). The study was reviewed and approved by the Institutional Review Board of Korea University Guro Hospital (IRB No. 2022GR0484).

### 2.2. Study Population

**Figure 3:**
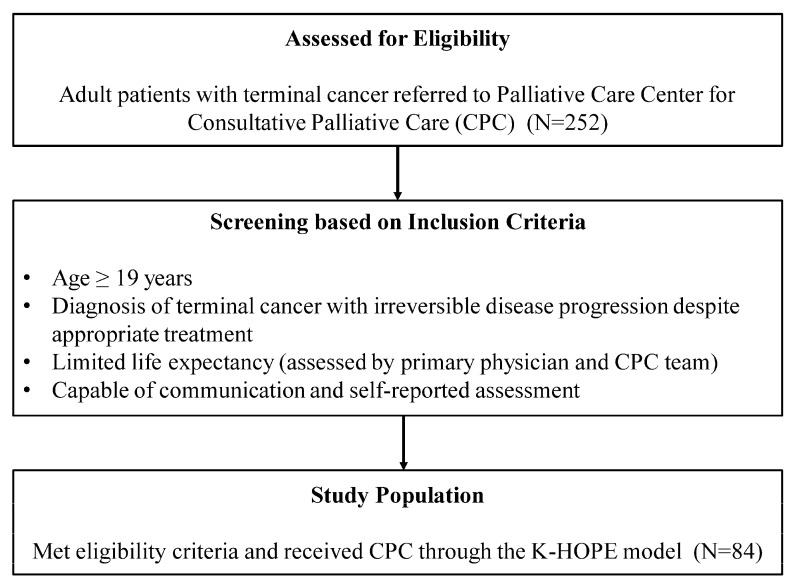
Eligibility and selection of the study population.

The study population consisted of adult patients with terminal cancer who were referred to the palliative care center for CPC. Terminal cancer was defined as advanced, incurable malignancies with irreversible disease progression despite appropriate disease-directed treatment and with an estimated life expectancy of several months, as determined by both the primary attending physicians and the CPC team. Patients were considered eligible if they were aged 19 years or older. Patients who were unable to communicate or complete self-reported assessments at the time of enrollment were excluded. During the study period, a total of 252 patients received CPC through the K-HOPE model. Among these, 84 patients consented to study participation and were included in the analysis (Figure 3).

### 2.3. Outcome Measures

Unmet needs related to physical symptoms, psychological distress, communication, information needs, and practical concerns were assessed using the Integrated Palliative care Outcome Scale (IPOS), a validated patient-reported outcome measure widely used in palliative care settings [15]. The IPOS comprises 10 questions with 17 scored items rated on a 5-point Likert scale (0–4), yielding a total score ranging from 0 to 68, with higher scores indicating greater burden and unmet needs [16]. Changes in total and domain-specific IPOS scores over time were used to evaluate longitudinal changes in multidimensional care needs during CPC involvement.

For patients who died during hospitalization, quality of death was evaluated using the Good Death Scale (GDS), which evaluates awareness and acceptance of death, respect for patient wishes, appropriateness of timing of death, and physical comfort during the final days of life [17]. Total scores range from 0 to 15. Consistent with prior studies, a GDS score of ≥12 was defined as indicating a good quality of death [18].

### 2.4. Statistical Analysis

Baseline demographic and clinical characteristics were summarized using descriptive statistics. Continuous variables are presented as means with standard deviations (SDs) or medians with interquartile ranges (IQRs), as appropriate. Categorical variables are presented as frequencies and percentages. Longitudinal changes in IPOS scores were analyzed using mixed-effects models for repeated measures (MMRMs), accounting for within-subject correlations across repeated assessments [19]. Time was included as a fixed effect, and individual patients as random effects. Estimated β coefficients with corresponding 95% confidence intervals (CIs) were reported. Patients were categorized into good-death and bad-death groups based on a GDS cutoff score. Between-group comparisons were performed using the chi-square test or Fisher’s exact test for categorical variables. Continuous variables were compared using the independent samples *t*-test or the Mann–Whitney U test, as appropriate, based on distributional assumptions. To identify factors independently associated with good death, multivariable logistic regression analysis was performed. Variables considered clinically relevant were entered into the model. Adjusted odds ratios (ORs) with 95% confidence intervals (CIs) were calculated. Statistical analyses were conducted using SPSS version 28.0 (IBM Corp., Armonk, NY, USA), with a two-sided *p*-value < 0.05 considered statistically significant.

## 3. Results

### 3.1. Characteristics of Participants

Demographic and clinical characteristics are summarized in Table 2. The mean age was 72.3 ± 7.1 years, and 57.1% of patients were male. Most patients were married (64.3%) and covered by the national health insurance system (84.5%). Hepatobiliary and pancreatic cancers were the most common primary diagnoses (37.0%), followed by lung cancer, malignant pleural mesothelioma (25.0%) and gastrointestinal cancers (14.3%). The majority of patients had received prior cancer-directed treatments, including chemotherapy (66.0%), surgery (48.8%), or radiotherapy (9.6%). At the time of CPC referral, 66.0% of patients had an Eastern Cooperative Oncology Group (ECOG) performance status of 4. The median duration of CPC involvement was 6 days (interquartile range [IQR], 3–9). Twenty-two patients (26.2%) died during hospitalization.

### 3.2. Serial Assessment of End-of-Life Care Domains Among Patients Receiving K-HOPE

Longitudinal changes in IPOS scores during CPC involvement were analyzed using mixed-effects models for repeated measures (MMRMs) (Table 3). The total IPOS score significantly decreased over time (β = −10.4, 95% confidence interval [CI], −12.8 to −8.0; *p* < 0.001), indicating a substantial reduction in overall symptom burden and unmet needs during CPC involvement. Domain-specific analyses demonstrated significant improvements in psychological and emotional distress (β = −2.4, 95% CI, −4.2 to −0.6; *p* = 0.010) and communication and information needs (β = −1.6, 95% CI, −2.4 to −0.8; *p* < 0.001). A reduction in physical symptom burden was observed (β = −2.0, 95% CI, −4.16 to 0.16; *p* = 0.069), although this did not reach statistical significance. Changes in practical and financial concerns were also not statistically significant (β = −0.3, 95% CI, −0.9 to 0.3; *p* = 0.310).

### 3.3. Quality of Death Among In-Hospital Decedents

Among the 22 patients who died during hospitalization, 13 patients (59.1%) were classified as having a good quality of death, defined as a GDS score ≥ 12 (Table 4). There were no statistically significant differences between the good-death and bad-death groups with respect to age, sex, marital status, health insurance type, educational level, religion, or ECOG performance status. However, the median duration of CPC involvement was significantly longer in the good-death group than in the bad-death group (7 days [interquartile range (IQR), 5–12] vs. 2 days [IQR, 1–3]; *p* = 0.015). Similarly, the duration of comfort care was significantly longer among patients in the good-death group 4 days [IQR, 4–9] vs. 1 day [IQR, 1–2]; *p* = 0.017).

### 3.4. Factors Independently Associated with Good Death

As shown in Table 5, a longer duration of CPC involvement was independently associated with higher odds of achieving a good death. Each additional day of CPC involvement was associated with a 42% increase in the odds of good death (adjusted OR, 1.42; 95% CI, 1.08–1.98; *p* = 0.021). Age and ECOG performance status were not significantly associated with quality of death in the multivariable model.

## 4. Discussion

In the Korean healthcare system, a substantial proportion of EOL care for patients with terminal cancer is delivered in acute-care hospitals, where fragmented workflows and limited time often hinder comprehensive palliative integration. In this context, the K-HOPE CPC model represents a structured and oncology-integrated approach designed to address multidimensional needs within routine hospital practice. Conceptualized as a model of palliative care delivery, CPC was evaluated using outcomes related to end-of-life care among hospitalized patients with terminal cancer. Our findings demonstrate meaningful improvements in key domains of EOL care, even within a relatively short period of involvement. In contrast to conventional CPC approaches, which are often implemented in heterogeneous ways across institutions, the K-HOPE model provides a structured and protocol-driven framework with clearly defined components and workflow. This standardization may enhance reproducibility and contribute to more consistent quality of palliative care delivery within the Korean healthcare system.

Using repeated patient-reported assessments, significant reductions in overall unmet needs were observed, with the most pronounced improvement in the communication and information domain, which reflects a core function of palliative care in supporting patient-centered communication at advanced stages of illness. This finding is clinically important, as communication regarding prognosis, goals of care, and EOL decision-making remains a persistent challenge in high-intensity cancer treatment environments [20]. Within oncology settings where disease-directed therapies often dominate clinical priorities, embedding structured family meetings and interdisciplinary communication processes into routine workflow may serve as an early and sensitive marker of effective palliative integration [21]. Consistent with prior evidence demonstrating that proactive communication in palliative care improves patients’ and families’ understanding and satisfaction, our findings suggest that standardized consultative frameworks can operationalize high-quality communication even in time-limited acute-care contexts, which is particularly relevant in the context of EOL care [22,23].

In contrast, changes in physical symptom burden and practical or financial concerns did not reach statistical significance. This pattern may reflect the clinical realities of tertiary oncology settings, where CPC is often initiated at a very advanced stage of illness [24]. Patients referred for CPC frequently experience rapidly evolving symptom trajectories driven by progressive disease, which may limit the extent of measurable short-term improvements despite active symptom management [25]. Moreover, practical concerns typically require sustained engagement, longitudinal communication, and continued support, which may be difficult to achieve during brief inpatient consultations. Previous studies have similarly reported that the impact of palliative care is often time-dependent, with greater benefits observed when palliative care is integrated earlier and maintained longitudinally across the disease trajectory [26,27].

Notably, the median duration of consultative palliative care (CPC) involvement was 6 days (IQR, 3–9), shorter than the national average of 8.2 days reported in Korea [14]. Although the study protocol planned a follow-up assessment three days after baseline and subsequent reassessments at seven-day intervals to capture changes in unmet needs more precisely, only 17 patients completed the first scheduled reassessment. Most patients died or were transferred to inpatient palliative care units, where longer-term care is available, before subsequent evaluations could be performed. This limited follow-up reflects a common challenge in tertiary oncology hospitals, where palliative care consultations are frequently initiated very late in the disease trajectory [28]. As a result, opportunities to evaluate longitudinal changes in patient-reported outcomes may be constrained in real-world inpatient settings. This pattern reflects the clinical context in Korea, where palliative care is often introduced late in the disease trajectory. Even within these constraints, short-term involvement was associated with meaningful improvements in patient-reported outcomes, while also suggesting the potential benefits of earlier and more proactive integration of palliative care. These findings are consistent with prior evidence. Prior systematic reviews indicate that earlier and sustained integration of palliative care is associated with greater improvements in quality of life, symptom control, and EOL care outcomes [29]. Similarly, evidence from large cohort analyses further suggests that earlier initiation of palliative care—well before the final months of life—is related to better EOL quality indicators, reinforcing the importance of proactive and timely referral [30].

Among patients who died during hospitalization, a longer duration of CPC involvement was significantly associated with higher quality of death. Patients in the good-death group had a substantially longer duration of CPC involvement than those in the bad-death group, and each additional day of CPC involvement increased the odds of achieving a good death in the multivariable analysis. These findings suggest that the continuity and duration of palliative care involvement, rather than patient demographic or clinical characteristics, may play a critical role in shaping EOL care [27]. In oncology settings where patients often experience rapid clinical decline near the EOL, even modest extensions in the duration of structured palliative care involvement provide meaningful opportunities for symptom management, communication, and preparation for death [10]. Previous studies have similarly reported that the timing of palliative care referral and consultation can influence EOL care outcomes. For example, earlier palliative care referrals in metastatic lung cancer were associated with longer hospice duration, lower frequency of aggressive interventions, and reduced rates of in-hospital death [31]. Furthermore, reviews of early palliative care models emphasize that sustained and timely integration of palliative care across the disease course is more likely to yield multidimensional benefits of EOL care than late or ad hoc consultation [32]. The GDS reflects multiple dimensions of a good death, including physical comfort, psychological acceptance, and alignment with patient preferences, highlighting the multidimensional nature of EOL care. The observed association between CPC duration and higher GDS scores suggests that structured palliative care interventions may influence several of these domains simultaneously. This further supports the need for a comprehensive and standardized model such as K-HOPE to address the complex and interrelated components of quality EOL care.

Several limitations of this study should be acknowledged. First, the duration of CPC involvement was relatively short, reflecting the acute-care hospital context and advanced disease status of the study population. This may have limited the ability to detect changes in domains such as psychological distress and practical concerns that typically require sustained engagement. Second, only patients able to complete self-reported assessments were included, which may have introduced selection bias [33]. In addition, although family support is a key component of the K-HOPE model, this study primarily focused on patient-reported outcomes and may not fully capture the broader impact on family caregivers, who often play a central role in EOL in Korea. Third, this study was conducted at a single tertiary hospital implementing a CPC model, which may limit the generalizability of the findings to other healthcare settings. Finally, the number of in-hospital decedents was relatively small. Although significant associations between CPC duration and quality of death were observed, these findings should be interpreted with caution.

Despite these limitations, our findings suggest that CPC integrated into tertiary acute-care hospitals can meaningfully improve key aspects of EOL care for patients with terminal cancer. The K-HOPE model demonstrates that structured, interdisciplinary palliative care embedded within routine inpatient oncology practice can enhance communication and address unmet needs without requiring dedicated palliative care units. These findings support the integration of palliative care as a routine component of oncology care rather than a service reserved solely for the final stage of life. At the policy level, these results highlight the need for healthcare systems to support earlier and more systematic integration of palliative care. In addition, educational efforts focusing on communication skills and interdisciplinary collaboration may further enhance the quality of EOL care. Future studies should focus on earlier referral and longitudinal models that extend across inpatient and outpatient settings to strengthen continuity of palliative care throughout the disease trajectory.

## 5. Conclusions

In conclusion, structured CPC, as a model of palliative care delivery, was associated with improvements in patient-reported outcomes related to EOL care among hospitalized patients with terminal cancer. Despite the short duration of involvement and late-stage referral, CPC demonstrated measurable benefits in communication and care experience, highlighting its potential role even in time-limited acute-care settings. These findings support the integration of palliative care into routine oncology practice and underscore the importance of earlier and more proactive implementation to maximize its impact on EOL care. Furthermore, a structured and standardized CPC framework may enhance the reproducibility and consistency of palliative care delivery within the Korean healthcare system.

## Figures and Tables

**Table 1:** Core components of the K-HOPE model.

Pre-Counseling
Information provision on palliative care
Advance care planning support
Holistic needs assessment and individualized care planning
Psychological, social, and spiritual support
Management of physical symptoms, including pain and other distressing symptoms
Family meetings, education, and psychosocial support
Financial support for socioeconomically disadvantaged patients
End-of-life care planning
Discharge planning and transition to inpatient palliative care or home-based services
Comfort care protocol implementation in the last days of life

Abbreviations: K-HOPE, Korea Holistic Optimized Palliative care for End-of-life.

**Table 2:** Characteristics of participants (*N* = 84).

General Characteristics	*n* (%) or Mean ± SD/Median (IQR)
Age (years)	72.3 ± 7.1
Sex	
Male	48 (57.1)
Female	36 (42.9)
Marital status	
Married ^a^	54 (64.3)
Unmarried ^b^	30 (35.7)
Health insurance	
National health insurance	71 (84.5)
Medical aid	13 (15.5)
Educational level	
High school or above	63 (75.0)
Below high school	21 (25.0)
Religion	
Yes ^c^	32 (38.1)
No	52 (61.9)
Clinical characteristics	
Primary cancer	
Hepatobiliary and pancreatic cancer	31 (37.0)
Lung and malignant pleural mesothelioma	21 (25.0)
Gastrointestinal cancer	12 (14.3)
Gynecologic cancer	8 (9.6)
Head and neck cancer	5 (6.0)
Hematologic malignancy	3 (3.6)
Others ^d^	4 (4.8)
Cancer treatment history ^e^	
Chemotherapy	55 (66.0)
Surgery	41 (48.8)
Radiation therapy	8 (9.6)
None	16 (19.2)
ECOG performance status	
1–3	29 (34.5)
4	55 (65.5)
Duration of CPC involvement (days)	6 (3–9)
In-hospital death	
Yes	22 (26.2)
No	62 (73.8)

Data are presented as n (%) or mean ± standard deviation (SD), except for CPC duration, which is presented as median (interquartile range [IQR]). ^a^ Married, remarried, or cohabiting, ^b^ Unmarried, including never married, divorced, separated, or widowed, ^c^ Religion present, including Christianity, Catholicism, or Buddhism, ^d^ Includes breast cancer, prostate cancer, spinal tumors, and thymic carcinoma, ^e^ Patients who received multiple cancer treatments were counted in each corresponding treatment category, Abbreviations: SD, standard deviation; ECOG, Eastern Cooperative Oncology Group; CPC, Consultative palliative care.

**Table 3:** Serial assessment of end-of-life care domains among patients receiving K-HOPE (N = 84).

Domain	Baseline	First Follow-Up	Β (SE)	95% CI	*p*-Value
Total score	35.1 ± 6.3	24.3 ± 5.8	−10.4 (1.2)	−12.8–−8.0	<0.001
Physical symptom burden	20.1 ± 5.3	17.2 ± 3.4	−2.0 (1.1)	−4.16–0.16	0.069
Psychological/emotional distress	9.1 ± 3.2	6.5 ± 3.3	−2.4 (0.9)	−4.2–−0.6	0.010
Communication and information needs	3.1 ± 1.4	1.5 ± 1.2	−1.6 (0.4)	−2.4–−0.8	<0.001
Practical and financial concerns	2.3 ± 1.2	2.0 ± 0.9	−0.3 (0.3)	−0.9–0.3	0.310

Data are presented as mean ± standard deviation (SD). Longitudinal changes were analyzed using mixed-effects models for repeated measures (MMRMs), with time included as a fixed effect and individual patients treated as random effects. β coefficients represent the estimated mean change from baseline to first follow-up with corresponding 95% confidence intervals (CIs). Abbreviations: K-HOPE, Korea Holistic Optimized Palliative Care for End-of-Life; SD, standard deviation; CI, confidence interval.

**Table 4:** Quality of death among in-hospital decedents receiving K-HOPE (*n* = 22).

		Bad Death Group (GDS < 12) (*n* = 9)	Good Death Group (GDS ≥ 12) (*n* = 13)	*p*-Value
Age (years), mean ± SD		70.3 ± 11.2	60.1 ± 6.5	0.355
Sex				0.758
Male		5 (55.6)	7(53.8)	
Female		4 (44.4)	6(46.2)	
Marital status				0.975
Married ^a^		5 (55.6)	8(61.5)	
Unmarried ^b^		4 (33.3)	5(38.5)	
Health insurance				0.253
National health insurance		7 (77.8)	13(100)	
Medical aid		2 (22.2)	0(0)	
Educational level				0.324
High school or above		8 (88.9)	12(92.3)	
Below high school		1 (11.1)	1(7.7)	
Religion				0.463
Yes ^c^		4 (44.4)	4 (30.8)	
No		5 (55.6)	9 (69.2)	
ECOG				1.000
3		0 (0.0)	2 (15.4)	
4		9 (100.0)	11 (84.6)	
Duration of CPC involvement (days), median (IQR)		2 (1–3)	7 (5–12)	0.015
Duration of comfort care (days), median (IQR)		1 (1–2)	4 (4–9)	0.017

Data are presented as mean ± standard deviation (SD) or number (%), unless otherwise indicated. Duration variables are presented as median (interquartile range [IQR]). Between-group comparisons were performed using the chi-square test or Fisher’s exact test for categorical variables and the independent *t*-test or Mann–Whitney U test for continuous variables, as appropriate. ^a^ Married, remarried, or cohabiting, ^b^ Unmarried, including never married, divorced, separated, or widowed, ^c^ Religion present, including Christianity, Catholicism, or Buddhism, Abbreviations: K-HOPE, Korea Holistic Optimized Palliative care for End-of-life; GDS, Good Death Scale; ECOG, Eastern Cooperative Oncology Group; CPC, consultative palliative care; SD, standard deviation; IQR, interquartile range.

**Table 5:** Multivariable logistic regression analysis for good death (n = 22).

Variable	Adjusted OR	95% CI	*p*-Value
Duration of CPC involvement (per 1–day increase)	1.42	1.08–1.98	0.021
Age (per 1–year increase)	0.97	0.89–1.06	0.482
ECOG 4 (vs. 3)	0.61	0.08–4.92	0.635

Multivariable logistic regression analysis was performed to identify factors independently associated with good death (defined as GDS ≥ 12). Adjusted odds ratios (ORs) with corresponding 95% confidence intervals (CIs) are presented. Abbreviations: GDS, Good Death Scale; OR, odds ratio; CI, confidence interval; ECOG, Eastern Cooperative Oncology Group; CPC, consultative palliative care.

## Data Availability

The data presented in this study are not publicly available due to privacy and ethical restrictions but are available from the corresponding author upon reasonable request.
